# Editorial: Arts and design in public health in a digital age: a multidisciplinary perspective

**DOI:** 10.3389/fpubh.2024.1485219

**Published:** 2024-09-23

**Authors:** Yao Song, Yan Luximon

**Affiliations:** ^1^School of Design, Hong Kong Polytechnic University, Kowloon, Hong Kong SAR, China; ^2^College of Literature and Journalism, Sichuan University, Chengdu, Sichuan, China

**Keywords:** arts, humanities, digital, artificial intelligence, public

In the current digital age, public health has embraced a more holistic approach, recognizing the critical role of social and technological factors in shaping health outcomes. This paradigm shift has led to a burgeoning interest in how arts and design can enhance public health initiatives by creating environments, interventions, and communication tools that foster healthy behaviors and improve overall wellbeing.

## The role of arts and design in public health

A growing body of research underscores the potential of art-based interventions in public health. Studies reveal that engagement with the arts can significantly improve mental health by alleviating stress, anxiety, and depression. Arts and design also promote social connections and foster health awareness, as seen in effective public health campaigns encouraging vaccinations and safe health practices. These interventions are not only about aesthetics but about creating impactful experiences that resonate with individuals and communities.

Artificial intelligence, including new technologies like social robots, is transforming public health education and intervention strategies (1, 2). AI can interact with cultural elements, digital technology, and user-centered design to deliver tailored and engaging health interventions.

AI-driven tools can be tailored to incorporate cultural elements, ensuring that health interventions are relevant and respectful of diverse traditions and practices. By understanding cultural contexts, AI can deliver messages that resonate more deeply with specific communities, enhancing the effectiveness of public health campaigns.

Digital technology enables the creation of interactive and adaptive learning environments. Online videos can simulate real-world scenarios, providing daily experiences that help individuals practice healthy behaviors. These technologies can make health education more engaging and memorable, leading to better retention and application of knowledge.

AI allows for the customization of health interventions based on user data. By analyzing user preferences and behaviors, AI can design personalized health plans that cater to individual needs. This user-oriented approach ensures that health interventions are not only effective but also more likely to be embraced by users, leading to sustained health improvements.

## Highlights from the Research Topic

This Research Topic presents a collection of original research articles, reviews, and opinion pieces that explore the intersection of arts, design, and public health:

The study by Xu et al. found that online short videos can significantly improve public knowledge about breast cancer symptoms, risk factors and screening methods, as well as positively influence attitudes toward breast self-examination.

Hearst et al.'s research demonstrated that engagement with local arts initiatives in Addis Ababa helped foster cultural understanding, reduce stigma around mental illness, and improve community support for those struggling with mental health issues.

Luo et al. developed a public first aid education model centered on user experience design principles, leading to improved knowledge retention, skill acquisition and motivation for first aid training among participants compared to traditional methods.

The study by Davies et al. found a positive association between recreational arts engagement (music, dance, visual arts) and better self-reported general health, reduced depression and higher life satisfaction in a cohort of older Australian adults.

Xuan et al.'s research showed that well-designed health science popularization videos can significantly enhance users' perceived usefulness, enjoyment and continuous usage intention of such content by increasing immersion and personal relevance.

Through computational analysis, Xue et al. uncovered distinct emotional patterns in religion-related films compared to other acclaimed movies, suggesting these films may provide a contemplative space aligning with spiritual themes to enable personal growth.

Urich et al.'s community campaign integrated arts with health messaging, leading to increased knowledge about preventing illnesses like diabetes and dementia, as well as high engagement from Hispanic community members.

Wang and Li explored how AI technologies like intelligent tutoring systems and virtual reality can provide personalized, immersive and adaptive learning experiences to improve public health education outcomes and accessibility.

## Future directions

The integration of arts and design in public health is a promising area for further research and innovation. There is a need for specialized design practices and multi-level art-based interventions to address public health challenges effectively. By exploring innovative solutions, we can better measure, assess, and improve health outcomes, reinforcing the significance of arts and design in this field.

Future research should focus on developing metrics to evaluate the impact of arts and design on health outcomes. Interdisciplinary collaboration between health professionals, artists, and designers can lead to innovative solutions tailored to specific community needs. Exploring the use of technology, such as virtual reality and artificial intelligence, can also enhance the delivery and impact of art-based interventions.

This Research Topic aims to substantiate the critical role of arts and design in public health, encouraging continued exploration and collaboration across disciplines to foster healthier communities in our digital age. By embracing the creative potential of arts and design, we can unlock new pathways to enhance public health and wellbeing.
